# StainCUT: Stain Normalization with Contrastive Learning

**DOI:** 10.3390/jimaging8070202

**Published:** 2022-07-20

**Authors:** José Carlos Gutiérrez Pérez, Daniel Otero Baguer, Peter Maass

**Affiliations:** Center for Industrial Mathematics, University of Bremen, 28359 Bremen, Germany; otero@uni-bremen.de (D.O.B.); pmaass@uni-bremen.de (P.M.)

**Keywords:** stain normalization, generative adversarial network, contrastive learning, digital pathology

## Abstract

In recent years, numerous deep-learning approaches have been developed for the analysis of histopathology Whole Slide Images (WSI). A recurrent issue is the lack of generalization ability of a model that has been trained with images of one laboratory and then used to analyze images of a different laboratory. This occurs mainly due to the use of different scanners, laboratory procedures, and staining variations. This can produce strong color differences, which change not only the characteristics of the image, such as the contrast, brightness, and saturation, but also create more complex style variations. In this paper, we present a deep-learning solution based on contrastive learning to transfer from one staining style to another: StainCUT. This method eliminates the need to choose a reference frame and does not need paired images with different staining to learn the mapping between the stain distributions. Additionally, it does not rely on the CycleGAN approach, which makes the method efficient in terms of memory consumption and running time. We evaluate the model using two datasets that consist of the same specimens digitized with two different scanners. We also apply it as a preprocessing step for the semantic segmentation of metastases in lymph nodes. The model was trained on data from one of the laboratories and evaluated on data from another. The results validate the hypothesis that stain normalization indeed improves the performance of the model. Finally, we also investigate and compare the application of the stain normalization step during the training of the model and at inference.

## 1. Introduction

In recent years, numerous deep-learning methods have been proposed to create Computer-Aided Diagnostic (CAD) systems to assist histopathologists [1,2,3]. These methods are trained using digital glass slides, known as Whole Slide Images (WSI), from one or more laboratories. The ultimate goal is to learn to generalize and perform well on images obtained from different laboratory environments including those that were not used during training.

One crucial step in pathological tissue preparation is the staining process, where dyes alter the intensity of tissue elements to make cellular structures distinguishable. The most common stain is Hemotoxylin and Eosin (H&E), where the hemotoxylin gives cell nuclei a blue or purple appearance and the eosin gives a pinkish hue to the cytoplasm and the extracellular matrix [4,5].

**Figure 1:**
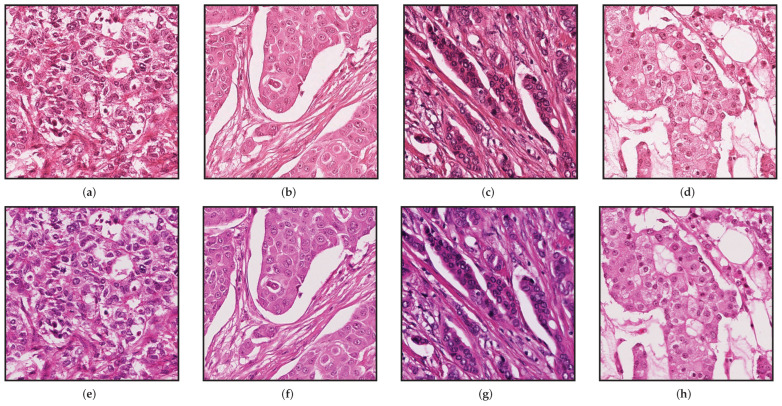
Patches at resolution 20×. (**a**–**d**) From Aperio Scanner and (**e**–**h**) from Hamamatsu Scanner.

There are many variables in the process of staining that change the appearance of the same tissue [6], for example, the concentration of the stain, time, manufacturer, and temperature at which the stain is applied. However, this process of staining is not the only source of variability in tissues, the digitization process can also introduce changes and variability in the tissue appearance. For example, Figure 1 shows the same physical specimen scanned using two different scanners. Pathologists are trained to be able to cope with those staining variations, for deep-learning methods, it is typically more difficult to cope with variations of staining and image appearance [7]. Therefore, preprocessing the input images to have the same appearance can potentially increase stability and robustness.

### Summary of Contributions

In this paper, we introduce a new deep-learning-based method for stain normalization of histopathological images. Our approach produces images with high similarity to the target domain and is inspired by the work of Park et al. [8] (contrastive learning for image–to–image translation). In Section 2, we present an overview of other existing stain normalization approaches.

We describe the architecture and contrastive learning-based training of our method in Section 3. In Section 4, we evaluate and compare against several state-of-the-art methods by using two datasets that consist of the same specimens but are digitized with two different scanners. We use image registration to create ground truths for the evaluation and four different metrics to compare the results.

Additionally, in Section 5, we evaluate our method as a preprocessing step in a clinical use case for the segmentation of breast cancer metastases in lymph nodes. The experiment is also performed using some of the approaches from Section 4, and we compare the application of the stain normalization at two different stages—namely, during training and during inference. To the best of our knowledge, such a comparison has not yet been investigated in the literature. Finally, in Section 6, we discuss the results, and in Section 7, we present and analyze some limitations of the method. For all the results, we performed statistical tests to validate if the observed differences were statistically significant or not. All the tables for the obtained *p*-values can be found in the Appendix B and Appendix C.

## 2. Related Work

One of the first methods for stain normalization was proposed by Reinhard et al. [9]. The approach is based on the transfer of color between an image taken as a reference and a color varied image using the statistical mean and variance of the two images. This method transforms the images, in a way that the contrast of the source image is similar to the reference image, and the image is transformed to the CIELAB color space in which the stains cannot be separated. Each channel is treated independently for alignment. The drawbacks of this approach have been discussed in [10,11].

The algorithms proposed by Macenko et al. [12] find the stains vectors for each image, using the color present in the reference image. This stain separation method is based on the fact that the color of the pixels in a histopathology image is a linear combination of two stain vectors (Eosin and Hematoxylin), where the weights of both of them are non-negative. This approach has few parameters, and no optimizations are required.

Ruifrok et al. [13] presented a novel supervised Color Deconvolution method; this approach maps the color distribution of a stained image to a stained target image. This method preserves the information of the source image. It uses a linear transformation in the CIELAB color space to match the statistics of each color channel in the two images in that color space. Prior information is needed in this method to estimate the color appearance matrix.

Khan et al. [10] proposed a method based on the nonlinear mapping of a source image to a target image using a representation derived from color deconvolution. A supervised color classification method, Relevance Vector Machine, is used to identify the locations where each stain is present. From these sets of classified pixels, the color appearance matrix and stain depth matrix are estimated. This method works at the pixel level and achieves a good result for stain separation.

The method by Vahadane et al. [14] for color normalization decomposes the image into a sparse and non-negative stain density map. This approach has two steps: stain separation by sparse nonnegative matrix factorization (SNMF), and structure–preserving color normalization. The sparseness added to the optimization equation of nonnegative matrix factorization helps to reduce the solution space; however, it increases the computational complexity. This approach preserves the structure of the source image; however, it does not preserve all the color variations of the source image, and the solution provided for the optimization problem may correspond to local minima rather than global minima.

Tam et al. [15] proposed a fully automated stain normalization method to reduce batch effects. They performed intensity centering and histogram equalization (ICHE) to normalize the intensity range of the image using centroid alignment. The image is divided into blocks, and each block’s intensity histogram is mapped to the target histogram.

With the development of deep-learning techniques in recent years, generative modeling paradigms, such as generative adversarial network (GAN) [16] and variational autoencoder (VAE) [17], and some methods for stain normalization has been proposed that can learn non–linear approaches. Zanjani et al. [18] presented three unsupervised methods for stain normalization based on different deep generative models: GAN, VAE, and deep convolutional Gaussian mixture model (DCGMM). Their approach does not require any labeled data and can generate non–linear transformations of the original image staining. Experiments by the authors show that the best score of the DCGMM method outperforms state–of–the–art methods by a 10–15% in color constancy on a dataset with several stain variations.

StainGAN [19] uses CycleGAN [20] for one–to–one domain stain transfer. Their approach can be trained end–to–end, eliminating the need for an expert to choose a reference image. Their method provided an improvement in tumor classification when applied during preprocessing. In [21], the authors also used a CycleGAN [20] approach, with a modified generator, and some changes in the loss function. They were able to improve the stability and performance of the segmentation of renal histopathology on slides obtained from different centers. Their results show a significant improvement when stain normalization is used in conjunction with standard data augmentation.

Cycle consistency, used in CycleGAN [20], has the underlying assumption that the relation between the two domains is a bijection, which can be overly restrictive in some contexts, as indicated in [8]. The assumption that the transformed image contains all the information to recover the original one is not always true, and this may result in certain features of the original image being preserved in the transformed image. However, in the context of stain normalization, this might not necessarily be a disadvantage.

Ciompi et al. [22] also investigated the importance of stain normalization using the Macenko method as a preprocessing step for tissue classification, showing how it can increase the performance of the models they evaluated. In [23], the authors also implemented the CycleGAN approach and included it as a preprocessing step for their nuclei segmentation method.

## 3. StainCUT

In this section, we introduce a new method for stain transfer/normalization, which is based on a modification of the method by Park et al. [8] for unpaired image–to–image translation. Unpaired image–to–image translation is a class of vision problems where the goal is to find a mapping from an input domain X to an output domain Y. In our case, $X\subset R^{H\times W\times C}$ and $Y\subset R^{H\times W\times C}$ correspond to RGB images with two different stains, respectively. Since this method does not need paired images from both domains, we will work with two datasets X={x∈X} and Y={y∈Y} of unpaired images from both.

The generator function G:X→Y, will be split in two components: an encoder $G_{\mathrm{enc}}$ and a decoder $G_{\mathrm{dec}}$, which are applied in sequence to obtain an output image $\hat{y}=G_{\mathrm{dec}}\left(G_{\mathrm{enc}}\left(x\right)\right)$. In [8], the authors show that, with their method, the encoder learns to capture domain–invariant concepts, i.e., the “content”, and the decoder learns to map the representations learned by the encoder to synthesize domain–specific features, i.e., the “style”.

As introduced in [16], the adversarial loss

(1) $$L_{\mathrm{GAN}}\left(G,D,X,Y\right)=E_{y∼Y}\log D\left(y\right)+E_{x∼X}\log\left(1-D\left(G\left(x\right)\right)\right)$$

is used to force the output of the generator to look similar to the images from the target domain. Here, *D* is a neural network that outputs a single scalar. D(z) represents the probability that *z* came from *Y* rather than as output of *G*.

Since corresponding patches in *x* and G(x) need to share some content, the authors proposed a way to force the network to preserve it using a contrastive learning objective function. The idea of contrastive methods is to learn representations by enforcing similar elements to be equal and not similar elements to be different. The Noise Contrastive Estimator (NCE) is used to achieve that

(2) $$l_{NCE}\left(v,v^{+},v^{-}\right)=-\log\left[\frac{\exp(v\;\cdot \;v^{+}/\tau )}{\exp\left(v\;\cdot \;v^{+}/\tau \right)+\sum_{n=1}^{N}\exp\left(v\;\cdot \;v_{n}^{-}/\tau \right)}\right]$$

In the equation above, $v^{+}$ is a data point similar to *v*, which means that the observations *v* and $v^{+}$ are correlated, and the pair $\left(v,v^{+}\right)$ represents a positive example. $v^{-}$ is a vector of examples not similar to *v*, and each pair $\left(v,v_{n}^{-}\right)$ forms a negative example. The reason to have a set of *N* negatives is that empirical results have shown that having many negative samples is good to obtain better representations. The factor τ=0.07 is used to scale the cosine similarity given by the dot product of the representations. This similarity measure is responsible for reducing the difference between positive pairs and maximizing the difference between negative ones.

Some *L* layers in the generator encoder $G_{\mathrm{enc}}$ are selected, and their feature maps are passed through a multi–layer perceptron (MLP) with one hidden layer $H_{l}$, similar to the setting introduced in SimCLR [24]. The output of $H_{l}$ is a stack of features ${\left\{z_{l}\right\}}_{L}={\left\{H_{l}\left(G_{\mathrm{enc}}^{l}\left(x\right)\right)\right\}}_{L}$, where $G_{\mathrm{enc}}^{l}$ represents the output of the *l*–th layer that was selected. To force the encoder to learn representations where patches in the same position of *x* and G(x) preserve content, the authors used a patch–wise contrastive loss.

The idea is that since a spatial location in the output of each layer of the encoder corresponds to a downsampled patch of the original input, the same locations in $G_{\mathrm{enc}}^{l}\left(x\right)$ and $G_{\mathrm{enc}}^{l}\left(G\left(x\right)\right)$ should be similar, and non–similar if they are in different locations. To make this observation formal, let index each layer l∈{1,2,…,L} and the spatial locations $s\in \{1,2,\ldots ,S_{l}\}$ within the layer. For each position we, have a feature map $z_{l}^{s}\in R^{C_{l}}$, the rest of positions give us other features $z_{l}^{S\backslash s}\in R^{\left(S_{l}-1\right)\times C_{l}}$, being $C_{l}$ the number of output channels in the encoder *l*–th layer. In a similar way, we can encode the output image $\hat{y}=G\left(x\right)$ into ${\left\{{\hat{y}}_{l}\right\}}_{L}={\left\{H_{l}\left(G_{\mathrm{enc}}^{l}\left(\hat{y}\right)\right)\right\}}_{L}$.

The PatchNCE loss is introduced as

(3) $$L_{PatchNCE}\left(G,H,X\right)=E_{x∼X}\sum_{l=1}^{L}\sum_{s=1}^{S_{l}}l_{NCE}\left({\hat{z}}_{l}^{s},z_{l}^{s},z_{l}^{S\backslash s}\right)$$

This loss attempts to match corresponding input–output patches representations at a specific location using the remaining positions as negative samples for the contrastive loss. The final objective is that translated images need to share content at the same patches but also need to look realistic, and this is given by

(4) $$L_{\mathrm{GAN}}\left(G,D,X,Y\right)+\lambda_{X}L_{PatchNCE}\left(G,H,X\right)+\lambda_{Y}L_{PatchNCE}\left(G,H,Y\right)$$

This method is known in the literature as Contrastive Unpaired Translation (CUT); therefore, we call our method for stain transfer based on contrastive learning: StainCUT.

### 3.1. Network Architecture

**Figure 2:**
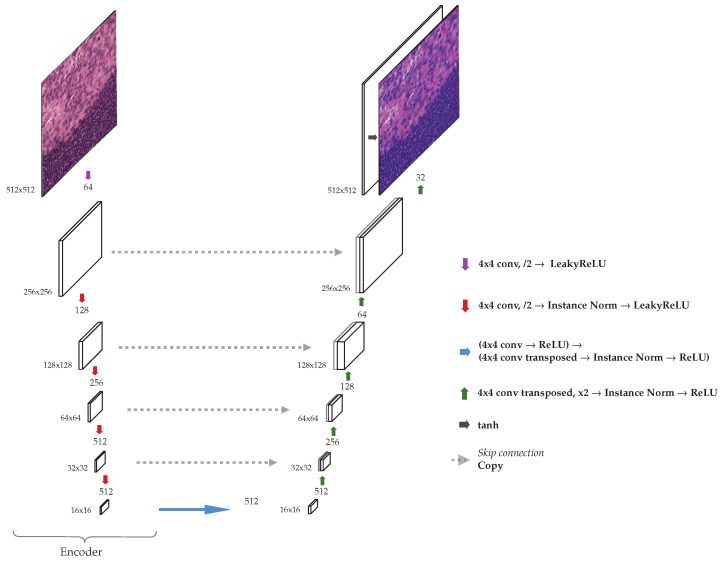
Modified UNet architecture used as a generator.

The generator we used is a modified UNet [25] (see Figure 2). The input is passed through an encoder, which downsamples the input image after each convolutional layer until a bottleneck is reached. The features encoded are then upsampled to generate an output image of the same dimensions as the original. Typical skip connections after each downsample are added, and these intermediate outputs are concatenated in a normal fashion in this type of architecture. Instead of using 3×3 convolutions two times, as in the original UNet [25], we only use a single convolutional layer for each downsampling operation, with a kernel–size of 4, a stride of 2, and padding of 1.

Each convolution applied reduces the size of the image by a factor of 2. After each convolution, an instance normalization layer was used, and after the normalization, LeakyRELU activation function with a negative slope equal to 0.2 was applied. For upsampling in the decoder transposed convolutions were used, with the same hyperparameters: kenel–size 4, stride 2, and padding 1. Furthermore, instance normalization layer and LeakyReLU were applied in each block in the decoder.

Before applying the encoder, a single convolution was applied to generate an image with 64 channels. After each encoder block, the number of channels is increased by a factor of 2, until a maximum possible of 512 channels, after which the number of channels stays the same. Each decoder block then reduces the number of channels by a factor of 2 accordingly after each step.

Since we applied a convolution to adjust the number of channels in the first layer of the encoder, the last layer of the decoder uses a transpose convolution in the same fashion to obtain a 3-channel output image. After this layer, a Tanh activation function was used. The maximum number of channels depends on the number of blocks selected for the network, in some of our experiments (Section 4) we use encoders with different numbers of blocks.

The discriminator used is a standard 70×70 PatchGAN introduced in [26] without any modification. For the calculation of the patch–wise contrastive loss, 256 random locations were sampled in each selected layer and a hidden layer MLP was applied to obtain a 256-dimension final features. Evenly distributed layers of the encoder in the generator were used to extract features for the MLP and PatchNCE loss computation.

## 4. Stain Transfer on the MITOS-ATYPIA Dataset

The goal of this experiment is to map images from slides scanned in different centers. The dataset used is publicly available as part of the MITOS-ATYPIA 14 challenge. It contains selected and annotated a set of breast cancer biopsy slides, each stained with hematoxylin and eosin (H&E) and scanned by two different scanners: Hamamatsu Nanozoomer 2.0-HT (Hamamatsu Photonics K.K., Hamamatsu City, Japan) and Aperio Scanscope XT (Leica Biosystems, Deer Park, TX, USA).

In every slide, the pathologist selected frames at magnification 20× located inside the tumor. The frames at 20× were divided into four frames at magnification 40×. The Aperio scanner has a resolution of 0.2455μm per pixel. The other scanner has a better resolution of 0.227299μm (horizontal) and 0.227531μm (vertical) per pixel, and thus a pixel is not exactly a square in the scanned slides. Detailed information about the resolution of both scanners and the size of the frames is given in Table 1.

### 4.1. Classic Methods

For comparison, four state–of–the–art methods were evaluated using public implementations: Macenko (https://warwick.ac.uk/fac/cross_fac/tia/software/sntoolbox/) (accessed on 13 July 2022) [12], Reinhard [9], Khan [10], and Vahadane (https://github.com/abhishekvahadane/CodeRelease_ColorNormalization) (accessed on 13 July 2022) [14].

**Figure 3:**
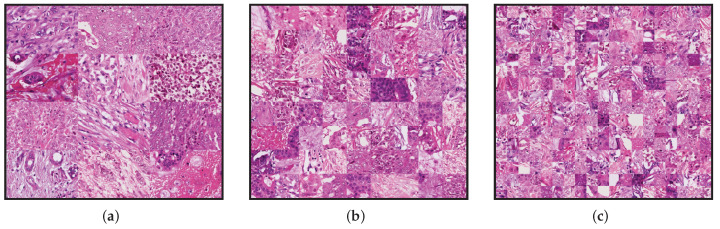
Downsampled template frames used in the classical methods. (**a**) Contains 3×4 tiles, (**b**) contains 9×8, and (**c**) contains 19×16 tiles. The size of the tiles in each template are 344×513 (**a**), 172×171 (**b**) and 86×81 (**c**), respectively.

We used three reference images as a template for those methods. All of these have the same dimensions as the original frames at 20× magnification and were generated by tiling patches extracted from different frames. The aim is to have a greater variety of color distribution across all the data present in the templates. The total number of tiles in each template is 3×4, 9×8, and 19×16, respectively. The size of the tiles in each template was calculated according to the dimension of the template size 1539×1376. These templates are shown in Figure 3.

### 4.2. Image Registration

For the experiments, we used all frames at resolution 20×. From those, we selected 300 for training, and the remaining 124 were used to test the performance of the method. Since each slide was scanned with two different scanners, not only the dimensions are different but also some misalignment due to rotation, and translation is present. For some of the metrics that we used, it is crucial to compare aligned images, in the sense that the content is the same at each position.

To handle this issue, we performed image registration using the MATLAB routine ‘imregtform’ (https://de.mathworks.com/help/images/ref/imregtform.html) (accessed on 13 July 2022), with affine transformations consisting of translation, rotation, scale, and shear. The optimization algorithm used for registration could handle well images with different brightness and contrast. After the registration, all frames have a size of 1539×1376. For the training set, we extracted 9000 random patches of size 600×600. The evaluation was performed at the frame level, using the 124 frames at 20× provided for the test. Figure 1 shows patches on the same positions in selected frames taken by the two scanners.

**Figure 4:**
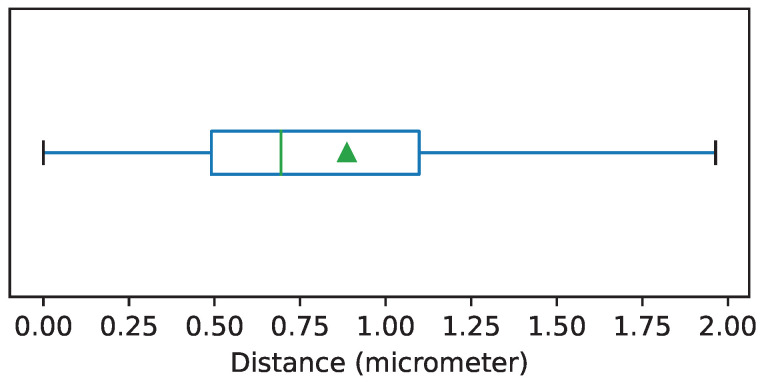
Boxplot showing the distribution of the average distances between control points after image registration. Each box is drawn from the first to the third quartile. The horizontal line represents the median value, and the triangle represents the mean. The whiskers indicate the minimum and maximum value of the distribution.

To evaluate the accuracy of the automatic registration method, we selected a random sample of 36 frames and register them manually using control points. For each case, the registration method creates a transformation matrix of dimensions 3×3. To compare the automatic and the manual registration we computed the average of the distances (in μm) between the locations, after registration, of the control points from the source image and their correct position on the target image. The mean of the distances was 0.8867μm with a standard deviation of 0.3377μm. The distribution of the distances is shown in Figure 4.

### 4.3. Training

We trained our model using three generators with the architecture as described in Section 3.1 and three different complexities: 4, 6, and 8 down-sampling blocks. In the rest of the manuscript, we refer to them as StainCUT UNet 4B, 6B, and 8B, respectively. For comparison, we also evaluated StainGAN [19] using the implementation of the authors (https://xtarx.github.io/StainGAN/) (accessed on 13 July 2022). Table 2 shows the number of parameters and the number of floating point operations (FLOPs) performed in every forward pass for each of the models used as a generator, i.e., the discriminator networks are not considered here, and for the StainGAN only one generator is taken into account. As it can be observed in Table 2, despite having fewer parameters than most of the evaluated StainCUT generators, the StainGAN generator performs more operations.

The model was trained for 30 epochs, using a batch–size of 4 and the Adam optimizer with a learning rate of 0.0002. The patches were randomly cropped to size 512×512 in the training step to give as input to the networks. The computations were made in one NVIDIA GeForce RTX 3080 Graphics card with a memory size of 10GB, while the StainGAN generator has few parameters than our UNet generator, the memory consumption for training using the contrastive learning approach is less than the StainGAN consumption. This is because the CycleGAN uses two generators and two discriminator networks, whereas the StainCUT approach uses only one.

Training the StainGAN with images of size 512×512 in our graphics card was not possible due to insufficient memory, not even with batch size 1; however, it was possible with the StainCUT method with batch size 4. The StainGAN method was trained then on images of size 384×384 with a batch–size of 2, and the rest of the hyperparameters were the same as the ones used for the StainCUT training. In the StainGAN paper [19], the authors reported training the method with images of size 256×256 with a batch size of 4 in a graphics card with a memory size of 12 GB. The classical methods can stain images with the dimensions of the frames at 20× level of magnification; however, training the deep-learning approaches with images from that size was not possible due to insufficient memory in the graphics card.

For that reason, we trained the methods with smaller tiles and stained the frames following a local strategy, i.e, staining small tiles from the frame independently and reconstructing the whole frame accordingly. The detailed approach goes as follows: the original frame was split into overlapping tiles, and each of them was stained using the generator; the overlapping is necessary to avoid artifacts between neighboring tiles. Since each pixel of the original frames can be in several tiles, the final value of a pixel is a weighted average of the values in each of the tiles where the pixel is present. For example, if a pixel is contained in four tiles, and the values of the pixel in those stained tiles are $x_{1},x_{2},x_{3},x_{4}$; the final value of the pixel in the reconstruction will be:

$$x=\frac{w_{1}x_{1}+w_{2}x_{2}+w_{3}x_{3}+x_{4}x_{4}}{w_{1}+w_{2}+w_{3}+w_{4}}$$

where $w_{1},w_{2},w_{3},w_{4}$ are weights that measure how close to the border of the corresponding tile the pixel is, and thus the tiles were the pixel is closed to the center of the tile contribute more to the final value.

The proposed method can be used to stain images of any dimensions and does not require a graphics card. In Table 3, we can see the average time spent by every method to stain a single frame of our test dataset. Our method is almost 2× faster than the StainGAN at evaluation time in the GPU and almost 4× in the CPU.

### 4.4. Results on Stain Transfer

**Figure 5:**
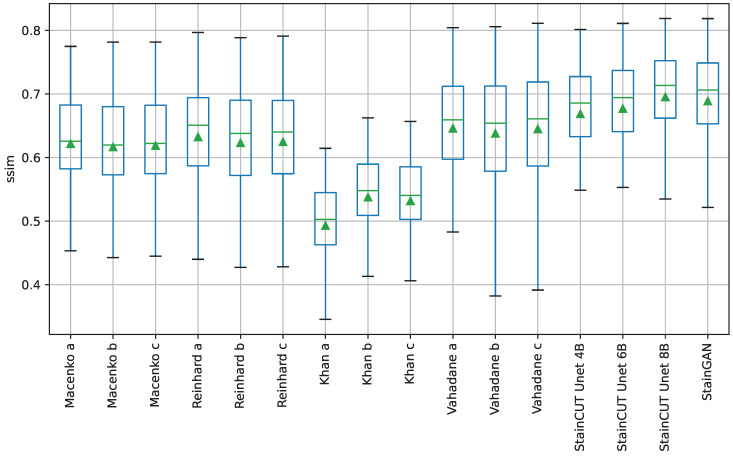
Boxplots from the evaluation of the SSIM metric for all compared methods on the MITOS-ATYPIA dataset. Each box is drawn from the first to the third quartile. The horizontal line represents the median value, and the triangle represents the mean. The whiskers indicate the minimum and maximum value of the distribution.

The performance of the methods was evaluated by comparing against the ground truths using four similarity measures: the Peak Signal-to-Noise Ratio (PSNR), Structural Similarity Index (SSIM) [27], Feature Similarity Index (FSIM) [28]) and the Learned Perceptual Image Patch Similarity (LPIPS) [29]. More information about these metrics can be found in Appendix A. The metrics’ results are provided in Table 4. Box-plots for the distribution of the results of the SSIM metric are depicted in Figure 5 and the ones for the other three metrics can be found in Appendix A. Finally, the results of the statistical tests are provided in Appendix B.

The statistical tests show that, in general, StainCUT with the different generators performs significantly different than Macenko, Reinhard, and Khan. However, when compared to Vahadane, there are cases when the more simple generators (UNet 4B and UNet 6B) do not give significantly different results. In the SSIM metric, StainCUT UNet 4B gives results not significantly different from Vahadane a (p-value=0.186), and Vahadane c (p-value=0.678). In the FSIM metric, StainCUT UNet 4B gives results not significantly different from Vahadane a (p-value=1.000), Vahadane b (p-value=1.000), and Vahadane c (p-value=1.000). In all other metrics, StainCUT has significantly different results than all the classical methods.

The tests show that there is no statistical difference between StainCUT UNet 6B and StainCUT UNet 8B with StainGAN. However, in the case of StainCUT UNet 4B, there are two metrics (SSIM and FSIM) where the results are significantly different from StainGAN.

**Figure 6:**
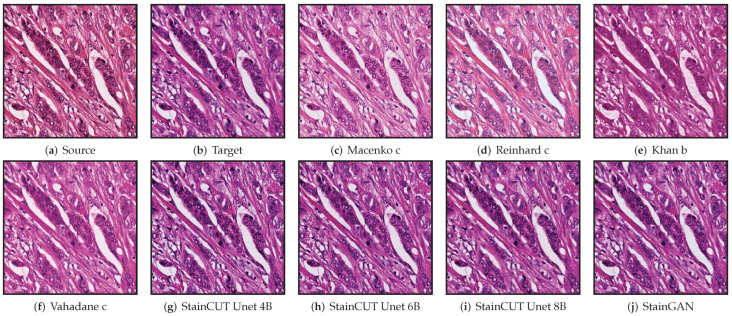
Stain normalization of source patch (**a**) to target patch (**b**) using different methods. For the classical methods, we show only the result the corresponds to the reference frame with the best SSIM score.

An example of the application of the compared methods is shown in Figure 6. There, one can observe that the StainCUT and StainGAN results are visually more similar to the ground truth than the ones obtained with the classic methods.

## 5. Use Case: Semantic Segmentation of Metastasis in Lymph Nodes

**Figure 7:**
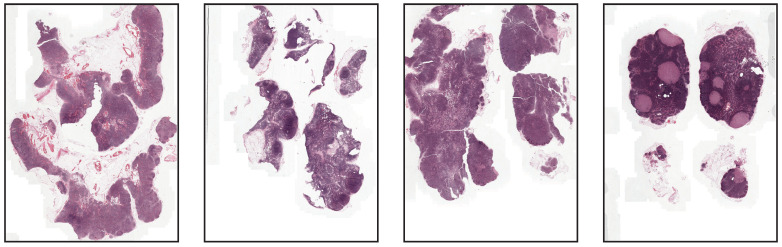
Low-resolution examples of whole-slide images from RUMC.

**Figure 8:**
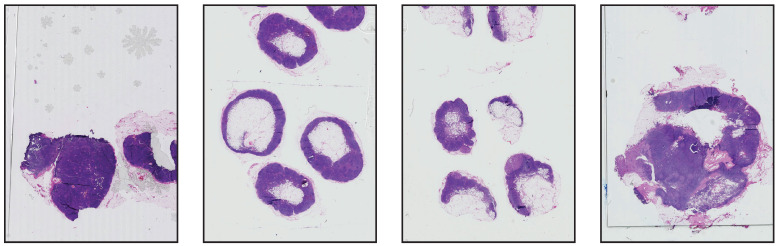
Low-resolution examples of whole-slide images from UMCU.

Our aim with this set of experiments is to evaluate the impact of using stain normalization in the context of semantic segmentation for the detection of breast cancer metastasis in lymph nodes. We trained a binary semantic segmentation model on WSIs from the CAMELYON16 (https://camelyon16.grand-challenge.org/) (accessed on 13 July 2022) dataset [30]. The WSIs come from two different medical centers in the Netherlands: Radboud University Medical Center (RUMC) and Utrecht University Medical Center (UMCU). An example of digitized slides from these centers can be seen in Figure 7 and Figure 8.

**Figure 9:**
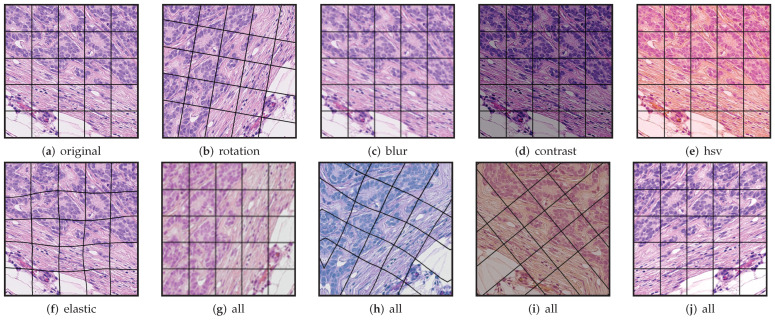
Augmentations used during training applied to a single patch.

We performed two experiments, and in each of them, we used the slides of one of the centers to train a segmentation network, whereas the other center was used for testing. To perform the binary semantic segmentation, a standard UNet [25] with a ResNet18 [31] encoder was used. The network was not pretrained with other data beforehand. To create the training dataset, we sampled patches at a magnification of 20×, with a size of 512 px × 512 px. In all cases, the network was trained using data augmentations, which included a composition of several elastic, flip, rotation, Gaussian blur and noise, fog, HSV color shift, brightness, and contrast transformation; see Figure 9.

Additionally, for each experiment, we used the stain transfer network in two different settings. In Setting (1), we performed the stain normalization during the training of the semantic segmentation network; i.e, we applied the stain normalization to the images used for training to make them look similar to the test images. In Setting (2), we performed the stain normalization to the test set at inference time; i.e, right before performing the inference with the segmentation model, we transform the images using the stain transfer method to make them look similar to the ones that were used for training. Setting (2) is what is typically found in the literature [22,23]; however, to the best of our knowledge, its comparison to Setting (1) has not yet been performed.

For each setting and each experiment, the stain transfer was performed with three different methods: Vahadane [14], StainGAN [19], and the introduced StainCUT approach. This way we are able to compare not only which method can better translate to another staining style but also their ability to capture and translate the essential features and whether it has an impact on the training and evaluation of a segmentation network.

The StainGAN and StainCUT methods were trained with the same hyper-parameters as described in Section 4. For the StainCUT, we used eight blocks in the generator, i.e., StainCUT UNet 8B. For the Vahadane method we used a mosaic of 4×4 tiles, each of size 128 px × 128 px. In Setting (1), the data augmentations were applied to the training patches after applying the stain transfer. Additionally, we trained the segmentation network using only data augmentations without any stain transfer to validate whether using the stain transfer indeed improves the performance of the model.

The data from each center was split into two datasets: one for the training of the stain normalization network and another for the training (when testing with the other center) and testing (when training with the other center) of the semantic segmentation task. To evaluate the segmentation, we used the dice coefficient corresponding to the tumor class, calculated using the probability heatmaps generated at magnification of 2.5× and the corresponding ground truth masks. The dice coefficient is calculated as:

$$DICE=\frac{2|X\cap Y|}{\left|X|+|Y\right|},$$

where *X* is the predicted set of pixels and *Y* is the ground truth.

### Results on Semantic Segmentation Use Case

**Figure 10:**
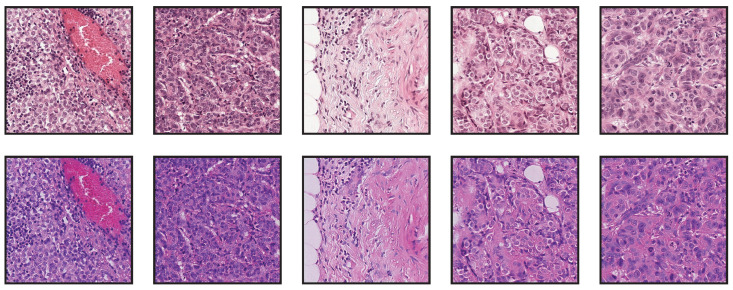
Exemplary tiles from the RUMC dataset (**top row**) and the transformed tiles after applying the stain normalization for the UMCU dataset (**bottom row**) using StainCUT.

**Figure 11:**
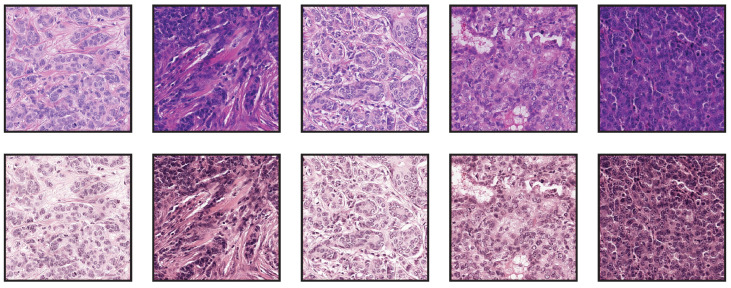
Exemplary tiles from the UMCU dataset (**top row**) and the transformed tiles after applying the stain normalization for the RUMC dataset (**bottom row**) using StainCUT.

In this section, we mainly present results on the performance of the segmentation model. In contrast to the dataset from Section 4, there are no ground truths for the stain transfer evaluation. Nevertheless, the quality of the stain transfer can be visually inspected. Some examples obtained with the StainCUT stain transfer approach are included in Figure 10 and Figure 11.

**Figure 12:**
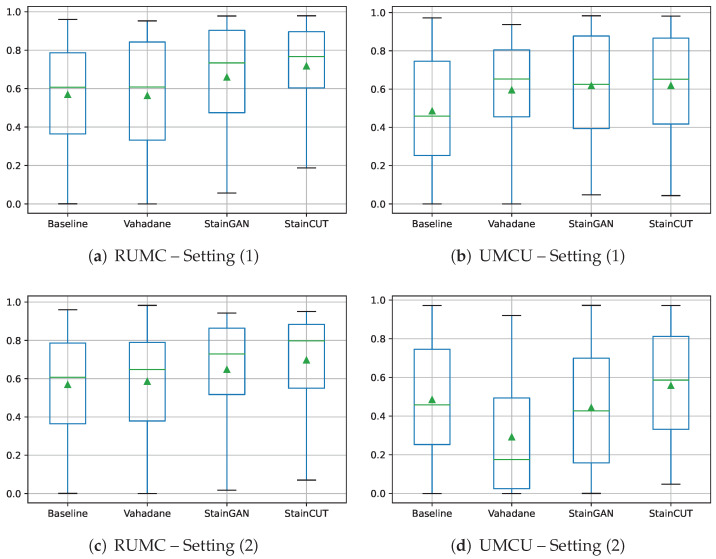
Distribution of the dice coefficient score for the segmentation of both datasets RUMC and UMCU, when the stain normalization is applied during training (Setting 1), and when stain normalization is applied during inference (Setting 2). The dice coefficient is calculated by comparing the generated segmentation masks and the ground truth masks for each WSI. In the boxplots, each box is drawn from the first to the third quartile. The horizontal line represents the median value, and the triangle represents the mean. The whiskers indicate the minimum and maximum value of the distribution.

The results of the semantic segmentation using Setting (1) are shown in Table 5, and using Setting (2) in Table 6. Each table shows the mean of the dice coefficient score from the tumor class calculated by comparing the generated masks and the ground truth masks. Box plots to visualize the distribution of those values are depicted in Figure 12. We also performed similar statistical tests to the ones from Section 4, and the results can be found in Appendix C.

Additionally, to determine if the application of the staining at training (Setting 1) or evaluation (Setting 2) time significantly affects the results, we performed a paired *t*-test [32] on the results for each of the compared methods; see Table A12 and Table A13 in Appendix C.

## 6. Discussion

In the MITOS-Atypia, the StainCUT approach with a UNet 8 Blocks generator had a better performance than all classic state-of-the-art methods (all $p-\mathrm{value}<10^{-3}$). This was further confirmed in the segmentation use case. The combination of the segmentation model with the StainCUT UNet 8 Blocks approach obtained significantly different results than the combination with Vahadane for both centers and both settings; see Table A8, Table A9, Table A10 and Table A11 (highest *p*-value was 0.046, which is still below the limit of 0.05).

One of the advantages of deep-learning-based methods, such as StainGAN and StainCUT, in comparison with the classic methods, is that they do not use a unique reference mosaic image, as those presented in Figure 3; they rather use a large dataset of images, which allows the models to better capture the distribution of the training data. Additionally, the method contains many parameters that are optimized by evaluating the performance of the model on the individual training images over several thousands of iterations. One can also adapt its complexity by varying the number of parameters. In the end, the model complexity allows the model to adapt the staining depending on the context or the patterns present in each of the input patches.

Although in all cases, the StainCUT approach obtained better results in the evaluated metrics than the StainGAN, both in the MITOS-Atypia dataset and in the semantic segmentation use case, in most of the cases there was not enough statistical evidence to claim that the results were significantly different. Nevertheless, in the segmentation use case with Setting (2), there was a significant difference in RUMC: 0.6475 vs. 0.6970 (p-value=0.013), and UMCU: 0.4439 vs. 0.5576 ($p-\mathrm{value}<10^{-3}$).

Additionally, the architecture of the generator used for StainCUT and the implemented contrastive learning approach provides a faster and more memory-efficient way to train and evaluate a stain normalization method. We believe that being able to train with larger patches does impact the quality of the model and its ability to better understand structures and patterns present in the image. Ideally, the model should learn how each of the different structures or types of nuclei present in histopathology images in the two different domains to be able to translate from one to the other. This also impacts the running time and in conjunction with a generator that performs a much lower number of FLOPs—see Table 2—results in a much more time-efficient method, as confirmed by our experiments; see Table 3.

The most important observation from the results of the semantic segmentation experiments—see Table 5, Table 6 and Table A8, Table A9, Table A10 and Table A11—is that, in the two analyzed settings, combining both stain normalization and color augmentation yields, in most of the cases, a statistically significant improvement over only using color augmentation: from 0.5684 to 0.7175 in RUMC ($p-\mathrm{value}<10^{-3}$) and from 0.4851 to 0.6178 in UMCU ($p-\mathrm{value}<10^{-3}$) with Setting (1); from 0.5684 to 0.6970 in RUMC ($p-\mathrm{value}<10^{-3}$) with Setting 2. For the UMCU center, in Setting 2, there was an improvement from 0.4851 to 0.5576; however, the p-value=0.076 indicates that the distribution of the errors is not significantly different. Nevertheless, we believe that the performed experiments confirm that it is a step that boosts the performance of the model and that using only color augmentations results in a sub-optimal performance.

The results also show that the phase at which the stain normalization is applied—during the training of the model (Setting 1) or at inference time (Setting 2)—makes a difference. In general, for the StainCUT method, the experiments using Setting (1) exhibit a higher mean dice coefficient score. The statistical tests show that the differences for the center RUMC when using StainCUT are not significant (see Table A12 (p-value=0.4639)); however, for the center UMCU, they are statistically significant for all the three compared methods; see Table A13 (all $p-\mathrm{values}<10^{-3}$).

In Setting (1), the segmentation network is being trained on data with a similar appearance to the data that will be used for inference, which might influence the way the model is trained. On the other hand, it has the disadvantage that the training process can be longer if we apply the staining just before passing the image to the segmentation network. This can be solved by staining all the images in the training dataset and storing them on the hard drive. Moreover, both the segmentation network and the stain normalization network have to be trained again for every different lab.

Setting (2) has the advantage that once the segmentation network has been trained, the only necessary step is to train the stain normalization network to transfer from the staining of the new images (from a different laboratory) to the staining of the images that were used during training. Therefore, Setting (2) is what is typically used in practice. Nevertheless, the results show it might be sub-optimal compared to Setting (1), i.e., using the stain normalization during training of the model might yield a better performance.

## 7. Limitations and Future Work

One of the main limitations of the deep-learning-based methods, such as StainGAN and StainCUT, is that there is no theoretical guarantee that the content will be preserved. The stain transfer is performed by the generator network, which was trained with a large dataset. However, if the input tile presents some anomaly or rare feature that was not present in the training data, it might happen that the result does not preserve the content of the image.

**Figure 13:**
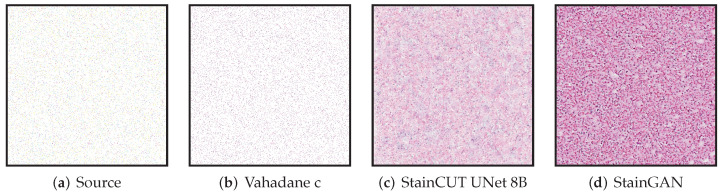
Example of applying the stain transfer (trained with the MITOS-Atypia dataset) to an out of distribution synthetic image (random salt and pepper noise with density 0.03). The result when applying the Vahadane method seems to be consistent; however, the StainCUT and StainGAN methods appear to attempt to create some tissue pattern.

**Figure 14:**
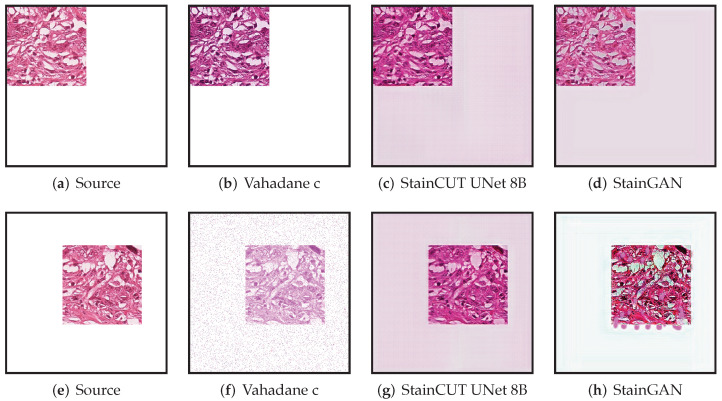
Example of applying the stain transfer (trained with the MITOS-Atypia dataset) to two out of distribution images. In the training dataset, all images were full of tissue. We have taken one of those images and made three quarters of it white. In the first case (**top row**), the result when applying the Vahadane method appears to be consistent; however, the StainCUT and StainGAN methods introduce a pink color in the white area. In the second case (**bottom row**), the StainCUT has a similar problem; however, Vahadane introduced some noise, and StainGAN produced some artifacts.

All though we did not find such a problem in any of the images we used for evaluating, the synthetic examples from Figure 13 and Figure 14 illustrate this behavior, where the StainCUT and StainGAN approaches have some issues. Even though it is a synthetic example, it is something that can happen; for example, in a WSI, the background is white, and thus there are many tiles that contain part of the tissue and part of the background. One could overcome this particular issue from the examples by also training the stain transfer with more images that contain a white background.

**Figure 15:**
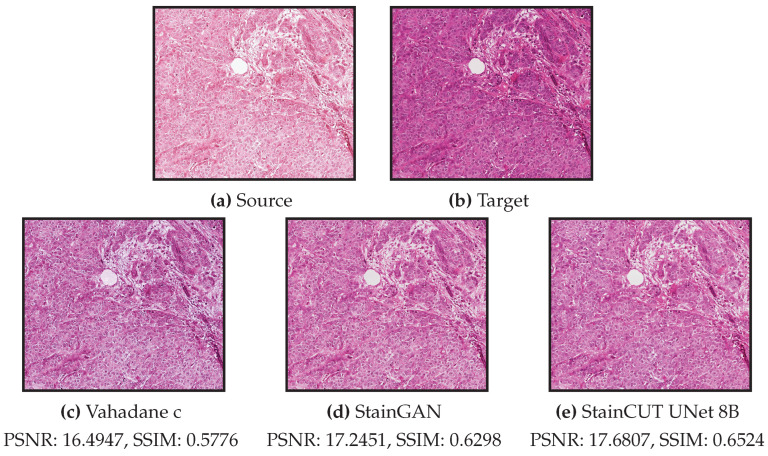
Exemplary source frame (**a**) where the stain transfer results (**c**–**e**) do not quite match the expected one (**b**). The content of the image is preserved in all cases. The images correspond to whole frames, i.e., the result frames are the result of combining several stained patches by the method described in Section 4.3.

In the experiments on the MITOS-ATYPIA dataset, the cases with low scores in the metrics are due to a mismatch with the target style. An example of such a case is depicted in Figure 15. There are several factors that could cause such behavior. If there are several stain variations within the training images of the target domain, in principle, transforming the source image to any of those variations would be correct. However, if the assigned ground truth (in this case the same image scanned with another scanner) exhibits another variation, the score will be low. Another factor could be that there were not enough images in the training set that correspond to the tissue patterns present in the source image.

Another limitation of the current algorithm is that it can only map from one staining style to another. However, it would be convenient to have a universal stain normalization method, that can bring any staining style, including new ones never seen, to a target reference staining style. This would mean that, for deploying the segmentation network, such as the one presented in Appendix C, to a new laboratory there is no need to train a new stain transfer network, which would reduce the complexity of the whole process. A future step would be to attempt to train the stain transfer network with data from many different staining styles, map to the reference style, and then evaluate if it works for an unseen staining style.

On the other hand, extending the method to work with other types of staining, such as immunostaining would only require using a training dataset with patches from that specific staining. However, the proposed method only aims to standardize images with different stain variations within the same staining type, e.g., H&E. A proper transfer from H&E to immunostaining is not possible since immunostaining relies on biological reactions that do not occur when staining with H&E. Nevertheless, transferring between H&E and immunostaining could be used as a technique to generate synthetic data.

## 8. Conclusions

We introduced a deep-learning-based method for solving the problem of stain normalization. The method was trained with unpaired images of two different laboratories or stain variations. It is based on the contrastive learning technique and a simplified UNet architecture. We evaluated its performance first in two different ways: using images of the same physical samples that were digitized with scanners from two different manufacturers and as part of the pipeline for the segmentation of metastasis of breast cancer in lymph nodes.

We also presented a realistic use-case application of our method—namely, the semantic segmentation of breast cancer metastases in lymph nodes. The model was trained with WSIs of one center and evaluated on WSIs of another center with a different staining variation. We trained the segmentation model using data augmentation in conjunction with (or without) stain normalization. The results show that stain normalization indeed boosts the performance in the two settings analyzed. Moreover, the results show that it might be more convenient to use the stain normalization step during the training of the model rather than at the inference time, which is what is typically done in the literature.

Our results show that the proposed method StainCUT with a UNet 8 Blocks generator outperformed classic state-of-the-art methods. It also achieved similar performance to another deep-learning-based approach: StainGAN, and in some of the experiments of the segmentation use case, the proposed method outperformed StainGAN with statistical significance. Additionally, the proposed method was faster to train and had a lower evaluation time than StainGAN despite having more parameters.

## Figures and Tables

**Table 1:** Resolution of the scanners and dimensions of frames on the MITOS-ATYPIA dataset.

	Aperio Scanscope XT	Hamamatsu Nanozoomer 2.0-HT
Resolution at 40×	0.2455μm per pixel	0.227299μm per pixel (horizontal) 0.227531μm per pixel (vertical)
Dimensions of a 20× frame	1539×1376 pixels 755.649×675.616μm${}^{2}$	1663×1485 pixels 755.9965×675.7671μm${}^{2}$
Dimensions of a 40× frame	1539×1376 pixels 377.8245×337.808μm${}^{2}$	1663×1485 pixels 337.9982×337.8835μm${}^{2}$

**Table 2:** Floating point operations (FLOPs) performed in every forward pass and number of parameters in the generators of the compared deep–learning architectures. The input size for the FLOPs computation was 512×512. For the StainGAN, only the FLOPs and the parameters that correspond to one of the generators are considered.

Model	FLOPs (G)	Params (M)
StainCUT UNet 4B	65.21	15.870
StainCUT UNet 6B	70.85	45.298
StainCUT UNet 8B	72.61	70.466
StainGAN	227.55	28.286

**Table 3:** Time per frame required by every method (mean ± std). For the deep-learning approaches, the time was measured using either only the CPU or both CPU and GPU. The methods that do not rely on neural networks were executed using only the CPU.

Method	Time (s/frame)
Macenko	1.103±0.076
Reinhard	1.034±0.104
Khan	106.018±2.104
Vahadane	18.359±1.437
StainCUT UNet 4B (CPU)	2.937±0.212
StainCUT UNet 6B (CPU)	3.024±0.077
StainCUT UNet 8B (CPU)	3.392±0.165
StainGAN (CPU)	12.731±0.392
StainCUT UNet 4B (GPU)	0.619±0.044
StainCUT UNet 6B (GPU)	0.637±0.009
StainCUT UNet 8B (GPU)	0.665±0.012
StainGAN (GPU)	1.107±0.054

**Table 4:** Evaluation metrics (mean ± std) for the stain transfer using the images from the Hamamatsu Scanner as reference. Bold highlights the best results for each metric.

Methods	SSIM	FSIM	PSNR	LPIPS
Macenko a	0.621±0.098	0.639±0.047	19.356±1.700	0.126±0.026
Macenko b	0.616±0.100	0.640±0.048	19.034±1.669	0.126±0.027
Macenko c	0.619±0.100	0.641±0.048	19.172±1.725	0.125±0.027
Reinhard a	0.632±0.102	0.641±0.047	19.309±1.731	0.114±0.023
Reinhard b	0.623±0.104	0.642±0.047	18.866±1.653	0.115±0.024
Reinhard c	0.625±0.104	0.641±0.047	18.999±1.717	0.114±0.024
Khan a	0.493±0.076	0.581±0.033	16.497±1.447	0.212±0.020
Khan b	0.537±0.079	0.600±0.036	17.638±1.458	0.195±0.018
Khan c	0.532±0.079	0.597±0.036	17.523±1.467	0.197±0.018
Vahadane a	0.646±0.105	0.647±0.050	18.783±2.120	0.114±0.031
Vahadane b	0.638±0.113	0.646±0.056	18.993±2.380	0.117±0.042
Vahadane c	0.645±0.111	0.648±0.055	19.268±2.401	0.113±0.041
StainCUT UNet 4B	0.669±0.097	0.652±0.047	20.686±2.056	0.092±0.021
StainCUT UNet 6B	0.677±0.098	0.658±0.048	20.575±1.961	0.090±0.020
StainCUT UNet 8B	0.695±0.096	0.663±0.046	21.176±2.036	0.083±0.019
StainGAN	0.689±0.098	0.666±0.047	20.754±1.949	0.084±0.019

**Table 5:** The mean values of the dice coefficient score for the segmentation of both datasets, when the stain normalization is applied during training (Setting 1). The dice coefficient is calculated comparing the generated masks and the ground truth masks for each WSI. The corresponding statistical tests can be found in Table A8 and Table A9 in Appendix C. Bold highlights the best results that are not significantly different.

Name	Stain Transformed	Dice Coefficient RUMC	Dice Coefficient UMCU
Baseline	✗	0.5684	0.4851
Vahadane	✓	0.5629	0.5950
StainGAN	✓	**0.6589**	**0.6176**
StainCUT	✓	**0.7175**	**0.6178**

**Table 6:** The mean values of the dice coefficient score for the segmentation of both datasets, when the stain normalization is applied during evaluation (Setting 2). The dice coefficient is calculated by comparing the generated masks and the ground truth masks for each WSI. The corresponding statistical tests can be found in Table A10 and Table A11 in Appendix C. Bold highlights the best results that are not significantly different.

Name	Stain Transformed	Dice Coefficient RUMC	Dice Coefficient UMCU
Baseline	✗	0.5684	**0.4851**
Vahadane	✓	0.5854	0.2919
StainGAN	✓	0.6475	0.4439
StainCUT	✓	**0.6970**	**0.5576**

**Figure A1:**
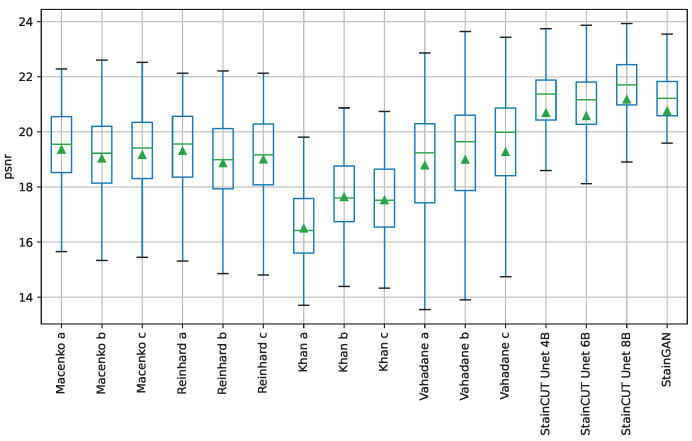
Boxplots of the evaluation of the PSNR metric for all compared methods on the MITOS-ATYPIA dataset. Each box is drawn from the first to the third quartile. The horizontal lines represent the median value, and the triangle represents the mean. The whiskers indicate the minimum and maximum value of the distribution.

**Figure A2:**
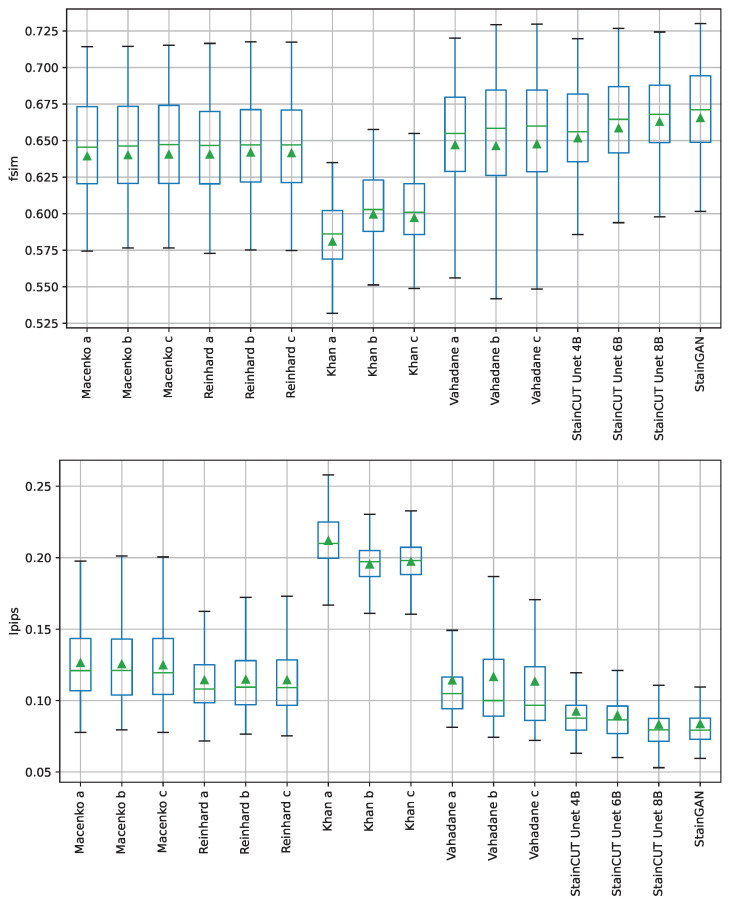
Boxplots of the evaluation of the FSIM and LPIPS metrics for all compared methods on the MITOS-ATYPIA dataset. Each box is drawn from the first to the third quartile. The horizontal lines represent the median value, and the triangle represents the mean. The whiskers indicate the minimum and maximum value of the distribution.

## Data Availability

The data presented in this study are available on request from the corresponding author.

## Appendix A. Metrics

In the following, we describe the metrics used in the results section:

Peak Signal-to-Noise Ratio (PSNR) is calculated as:

(A1) $$PSNR\left(x,y\right)=20\log_{10}\left(L\right)-10\log_{10}\left(MSE\left(x,y\right)\right)$$

where MSE(x,y) is the mean square error between the *x* and *y*, and *L* is the maximum possible pixel value of the image, i.e., L=255.

Structural similarity index measure (SSIM) [27] is a perceptually based metric and considers changes in structural information rather than calculating the absolute errors as in PSNR. Structural information is the idea that pixels have strong interdependencies, particularly when they are spatially close. The SSIM index is calculated on various windows of an image. The measure between two windows *x* and *y* of size n×n is:

(A2) $$SSIM\left(x,y\right)=\frac{\left(2\mu_{x}\mu_{y}+c_{1}\right)\left(2\sigma_{xy}+c_{2}\right)}{\left(\mu_{x}^{2}+\mu_{y}^{2}+c_{1}\right)\left(\sigma_{x}^{2}+\sigma_{y}^{2}+c_{2}\right)}$$

where

- $\mu_{x}$ is the average of the pixels in *x*,
- $\mu_{y}$ is the average of the pixels in *y*,
- $\sigma_{x}^{2}$ is the variance of the pixels in *x*,
- $\sigma_{y}^{2}$ is the variance of the pixels in *y*,
- $\sigma_{xy}$ is the covariance of the pixels in *x* and *y*,
- $c_{1}={\left(k_{1}L\right)}^{2}$ and $c_{2}={\left(k_{2}L\right)}^{2}$ are to two constants to stabilize the division,
- *L* is the maximum possible pixel value of the image, i.e., L=255, and
- $k_{1}=0.01$ and $k_{2}=0.03$ are fixed by default.

Additionally, we evaluated the Feature-based similarity index (FSIM) [28] and the Learned Perceptual Image Patch Similarity (LPIPS) [29] metrics.

All metrics, except for the LPIPS metric were evaluated using the ‘image-similarity-measures’ Python package (https://pypi.org/project/image-similarity-measures/) (accessed on 13 July 2022). For the LPIPS metric, we used the official repository (https://github.com/richzhang/PerceptualSimilarity) (accessed on 13 July 2022).

## Appendix B. Statistical Tests on the Metrics of the MITOS-ATYPIA Dataset

We performed statistical tests on each metric to validate the results. First, to determine if there are differences between the methods, we performed a Friedman test [33]. After the statistical test rejected the null hypothesis that the group measurements are similar, we performed the Nemenyi test [34] to find which groups differ. The significance level chosen was 0.05 in all cases.

**Table A1:** The Friedman rank sum test for each of the metrics used.

Metric	Chi-Square	df	*p*-Value
SSIM	1628.7	15	<$2.2\times 10^{-16}$
FSIM	1476.0	15	<$2.2\times 10^{-16}$
PSNR	1226.9	15	<$2.2\times 10^{-16}$
LPIPS	1560.4	15	<$2.2\times 10^{-16}$

**Table A2:** Rows/Columns corresponding to methods in the stain transfer experiment.

Method	Renamed Method
Macenko a	1
Macenko b	2
Macenko c	3
Reinhard a	4
Reinhard b	5
Reinhard c	6
Khan a	7
Khan b	8
Khan c	9
Vahadane a	10
Vahadane b	11
Vahadane c	12
StainCUT UNet 4B	13
StainCUT UNet 6B	14
StainCUT UNet 8B	15
StainGAN	16

**Table A3:** *p*-values obtained from the Nemenyi test applied to the SSIM results. Row/Column names are listed in Table A2.

	1	2	3	4	5	6	7	8	9	10	11	12	13	14	15
2	0.668	-	-	-	-	-	-	-	-	-	-	-	-	-	-
3	1.000	0.964	-	-	-	-	-	-	-	-	-	-	-	-	-
4	0.907	0.005	0.516	-	-	-	-	-	-	-	-	-	-	-	-
5	0.629	1.000	0.952	0.004	-	-	-	-	-	-	-	-	-	-	-
6	0.997	1.000	1.000	0.128	1.000	-	-	-	-	-	-	-	-	-	-
7	0.000	0.000	0.000	0.000	0.000	0.000	-	-	-	-	-	-	-	-	-
8	0.000	0.000	0.000	0.000	0.000	0.000	0.038	-	-	-	-	-	-	-	-
9	0.000	0.000	0.000	0.000	0.000	0.000	0.921	0.942	-	-	-	-	-	-	-
10	0.000	0.000	0.000	0.052	0.000	0.000	0.000	0.000	0.000	-	-	-	-	-	-
11	0.022	0.000	0.002	0.902	0.000	0.000	0.000	0.000	0.000	0.973	-	-	-	-	-
12	0.000	0.000	0.000	0.003	0.000	0.000	0.000	0.000	0.000	1.000	0.619	-	-	-	-
13	0.000	0.000	0.000	0.000	0.000	0.000	0.000	0.000	0.000	0.186	0.001	0.678	-	-	-
14	0.000	0.000	0.000	0.000	0.000	0.000	0.000	0.000	0.000	0.000	0.000	0.000	0.506	-	-
15	0.000	0.000	0.000	0.000	0.000	0.000	0.000	0.000	0.000	0.000	0.000	0.000	0.000	0.095	-
16	0.000	0.000	0.000	0.000	0.000	0.000	0.000	0.000	0.000	0.000	0.000	0.000	0.000	0.716	1.000

**Table A4:** *p*-values obtained from the Nemenyi test applied to the FSIM results. Row/Column names are listed in Table A2.

	1	2	3	4	5	6	7	8	9	10	11	12	13	14	15
2	0.980	-	-	-	-	-	-	-	-	-	-	-	-	-	-
3	0.361	0.999	-	-	-	-	-	-	-	-	-	-	-	-	-
4	1.000	0.973	0.326	-	-	-	-	-	-	-	-	-	-	-	-
5	0.034	0.810	1.000	0.028	-	-	-	-	-	-	-	-	-	-	-
6	0.861	1.000	1.000	0.833	0.966	-	-	-	-	-	-	-	-	-	-
7	0.000	0.000	0.000	0.000	0.000	0.000	-	-	-	-	-	-	-	-	-
8	0.000	0.000	0.000	0.000	0.000	0.000	0.018	-	-	-	-	-	-	-	-
9	0.000	0.000	0.000	0.000	0.000	0.000	0.847	0.930	-	-	-	-	-	-	-
10	0.000	0.000	0.005	0.000	0.114	0.000	0.000	0.000	0.000	-	-	-	-	-	-
11	0.000	0.000	0.000	0.000	0.002	0.000	0.000	0.000	0.000	1.000	-	-	-	-	-
12	0.000	0.000	0.000	0.000	0.000	0.000	0.000	0.000	0.000	0.803	1.000	-	-	-	-
13	0.000	0.000	0.000	0.000	0.003	0.000	0.000	0.000	0.000	1.000	1.000	1.000	-	-	-
14	0.000	0.000	0.000	0.000	0.000	0.000	0.000	0.000	0.000	0.000	0.001	0.052	0.001	-	-
15	0.000	0.000	0.000	0.000	0.000	0.000	0.000	0.000	0.000	0.000	0.000	0.000	0.000	0.847	-
16	0.000	0.000	0.000	0.000	0.000	0.000	0.000	0.000	0.000	0.000	0.000	0.000	0.000	0.081	0.996

**Table A5:** *p*-values obtained from the Nemenyi test applied to the PSNR results. Row/Column names are listed in Table A2.

	1	2	3	4	5	6	7	8	9	10	11	12	13	14	15
2	0.018	-	-	-	-	-	-	-	-	-	-	-	-	-	-
3	0.810	0.949	-	-	-	-	-	-	-	-	-	-	-	-	-
4	1.000	0.225	0.999	-	-	-	-	-	-	-	-	-	-	-	-
5	0.000	0.568	0.005	0.000	-	-	-	-	-	-	-	-	-	-	-
6	0.000	1.000	0.427	0.017	0.982	-	-	-	-	-	-	-	-	-	-
7	0.000	0.000	0.000	0.000	0.000	0.000	-	-	-	-	-	-	-	-	-
8	0.000	0.000	0.000	0.000	0.081	0.000	0.002	-	-	-	-	-	-	-	-
9	0.000	0.000	0.000	0.000	0.000	0.000	0.537	0.912	-	-	-	-	-	-	-
10	0.000	0.964	0.069	0.001	1.000	1.000	0.000	0.006	0.000	-	-	-	-	-	-
11	0.011	1.000	0.907	0.163	0.668	1.000	0.000	0.000	0.000	0.983	-	-	-	-	-
12	0.934	0.840	1.000	1.000	0.001	0.246	0.000	0.000	0.000	0.028	0.761	-	-	-	-
13	0.000	0.000	0.000	0.000	0.000	0.000	0.000	0.000	0.000	0.000	0.000	0.000	-	-	-
14	0.000	0.000	0.000	0.000	0.000	0.000	0.000	0.000	0.000	0.000	0.000	0.000	1.000	-	-
15	0.000	0.000	0.000	0.000	0.000	0.000	0.000	0.000	0.000	0.000	0.000	0.000	0.019	0.035	-
16	0.000	0.000	0.000	0.000	0.000	0.000	0.000	0.000	0.000	0.000	0.000	0.000	0.999	1.000	0.446

**Table A6:** *p*-values obtained from the Nemenyi test applied to the LPIPS results. Row/Column names are listed in Table A2.

	1	2	3	4	5	6	7	8	9	10	11	12	13	14	15
2	1.000	-	-	-	-	-	-	-	-	-	-	-	-	-	-
3	1.000	0.999	-	-	-	-	-	-	-	-	-	-	-	-	-
4	0.578	0.506	0.995	-	-	-	-	-	-	-	-	-	-	-	-
5	0.840	0.786	1.000	1.000	-	-	-	-	-	-	-	-	-	-	-
6	0.300	0.246	0.946	1.000	1.000	-	-	-	-	-	-	-	-	-	-
7	0.000	0.000	0.000	0.000	0.000	0.000	-	-	-	-	-	-	-	-	-
8	0.000	0.000	0.000	0.000	0.000	0.000	0.099	-	-	-	-	-	-	-	-
9	0.000	0.000	0.000	0.000	0.000	0.000	0.678	1.000	-	-	-	-	-	-	-
10	0.003	0.002	0.106	0.897	0.668	0.986	0.000	0.000	0.000	-	-	-	-	-	-
11	0.001	0.001	0.052	0.770	0.486	0.946	0.000	0.000	0.000	1.000	-	-	-	-	-
12	0.000	0.000	0.000	0.005	0.001	0.022	0.000	0.000	0.000	0.697	0.847	-	-	-	-
13	0.000	0.000	0.000	0.000	0.000	0.000	0.000	0.000	0.000	0.000	0.000	0.001	-	-	-
14	0.000	0.000	0.000	0.000	0.000	0.000	0.000	0.000	0.000	0.000	0.000	0.000	0.966	-	-
15	0.000	0.000	0.000	0.000	0.000	0.000	0.000	0.000	0.000	0.000	0.000	0.000	0.011	0.668	-
16	0.000	0.000	0.000	0.000	0.000	0.000	0.000	0.000	0.000	0.000	0.000	0.000	0.018	0.752	1.000

## Appendix C. Statistical Tests on the Semantic Segmentation Use–Case

**Table A7:** Friedman rank sum test for each of the settings and centers used.

Experiment	Chi-Square	df	*p*-Value
Setting (1) RUMC	33.39	3	$2.665\times 10^{-7}$
Setting (1) UMCU	50.04	3	$7.847\times 10^{-11}$
Setting (2) RUMC	36.93	3	$4.761\times 10^{-8}$
Setting (2) UMCU	101.96	3	<$2.2\times 10^{-16}$

**Table A8:** *p*-values obtained from the Nemenyi test applied to the RUMC with Setting (1).

	Baseline	Vahadane	StainGAN
Vahadane	0.998	-	-
StainGAN	0.037	0.022	-
StainCUT	0.000	0.000	0.160

**Table A9:** *p*-values obtained from the Nemenyi test applied to the UMCU with Setting (1).

	Baseline	Vahadane	StainGAN
Vahadane	0.039	-	-
StainGAN	0.000	0.001	-
StainCUT	0.000	0.046	0.605

**Table A10:** *p*-values obtained from the Nemenyi test applied to the RUMC with Setting (2).

	Baseline	Vahadane	StainGAN
Vahadane	1.000	-	-
StainGAN	0.133	0.110	-
StainCUT	0.000	0.000	0.013

**Table A11:** *p*-values obtained from the Nemenyi test applied to the UMCU with Setting (2).

	Baseline	Vahadane	StainGAN
Vahadane	0.000	-	-
StainGAN	0.182	0.000	-
StainCUT	0.076	0.000	0.000

**Table A12:** Paired *t*-test to compare Setting (1) to Setting (2) in center RUMC.

Experiment	t-Statistic	*p*-Value
Vahadane	−0.8746	0.3872
StainGAN	0.5864	0.5610
StainCUT	0.7398	0.4639

**Table A13:** Paired *t*-test to compare Setting (1) to Setting (2) in center UMCU.

Experiment	t-Statistic	*p*-Value
Vahadane	9.0977	$1.7507\times 10^{-13}$
StainGAN	8.5734	$1.6063\times 10^{-12}$
StainCUT	3.4820	$8.6176\times 10^{-4}$
